# Supplementary material for: Genome-Wide Identification of CHYR Gene Family in Sophora alopecuroides and Functional Analysis of SaCHYR4 in Response to Abiotic Stress
Source: Int J Mol Sci. 2024 Jun 4;25(11):6173. doi: 10.3390/ijms25116173 (PMC11173228; doi:10.3390/ijms25116173)
Supplement: Supplementary file 1 [file ijms-25-06173-s001.zip › Supplementary_Materials.pdf]

Table S1. Primers used for detection and real-time PCR analyses

Primer name	Primer sequence (5′ to 3′)
SaCHYR4-F	GGAATTCTCAGCTTGTGATATG
SaCHYR4-R	AACTGCAGTCAGCCTCTTGTTTG
J-*SaCHYR4*-F	CCTCACCCTCACTTAT
J-*SaCHYR4*-R	AAAACAGGCACTCAAA
q-*SaCHYR4*-F	GCTGTTCTCTTGCATGGTGT
q-*SaCHYR4*-R	ACACACGACAAACAAGAGGC
Lectin-F	GTCCTTTCAGGAGGTACAACC
Lectin-R	CCACATCTGCTGGAAGGTGC

Table S3. The predication scores of protein structures

No.	Protein name	QMEANDisCo
1	SaCHYR1	0.75
2	SaCHYR2	0.91
3	SaCHYR3	0.85
4	SaCHYR4	0.85
5	SaCHYR5	0.72
6	SaCHYR6	0.72
7	SaCHYR7	0.91
8	SaCHYR8	0.91
9	SaCHYR9	0.94
10	SaCHYR10	0.94
11	SaCHYR11	0.73
12	SaCHYR12	0.73
13	SaCHYR13	0.85
14	SaCHYR14	0.85
15	SaCHYR15	0.72
16	SaCHYR16	0.72
17	SaCHYR17	0.93
18	SaCHYR18	0.87

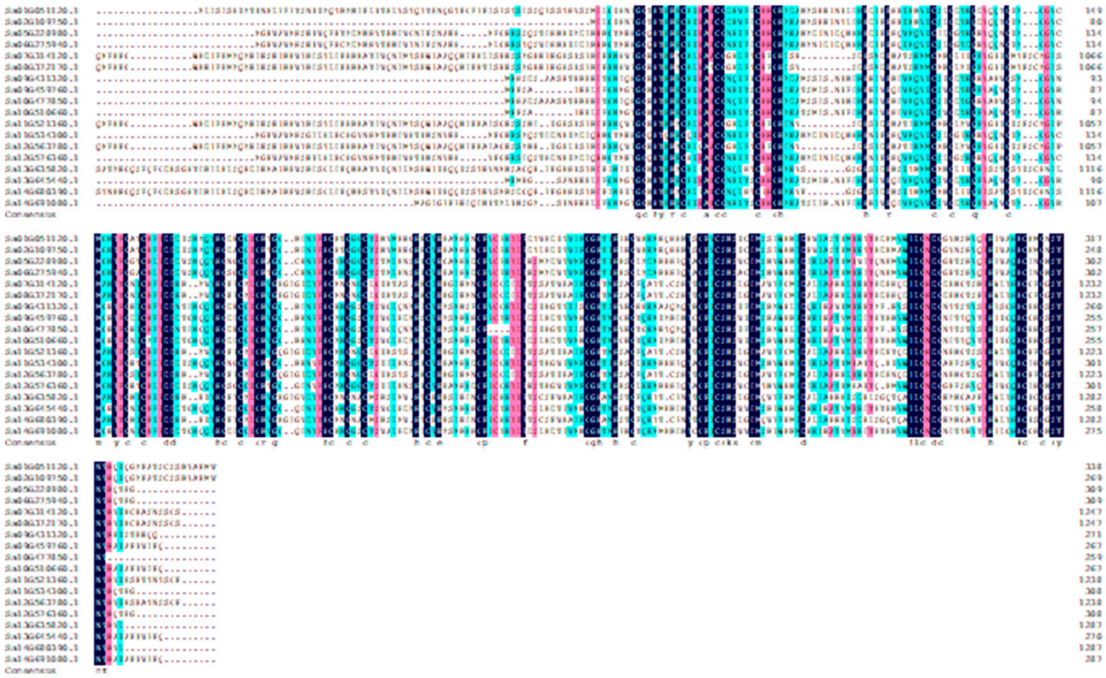

Figure S1. Multiple sequence alignments of zf-CHY, Ring H2, and zinc ribbon_6 conserved domains in all CHYR proteins, as analyzed using DNAMAN

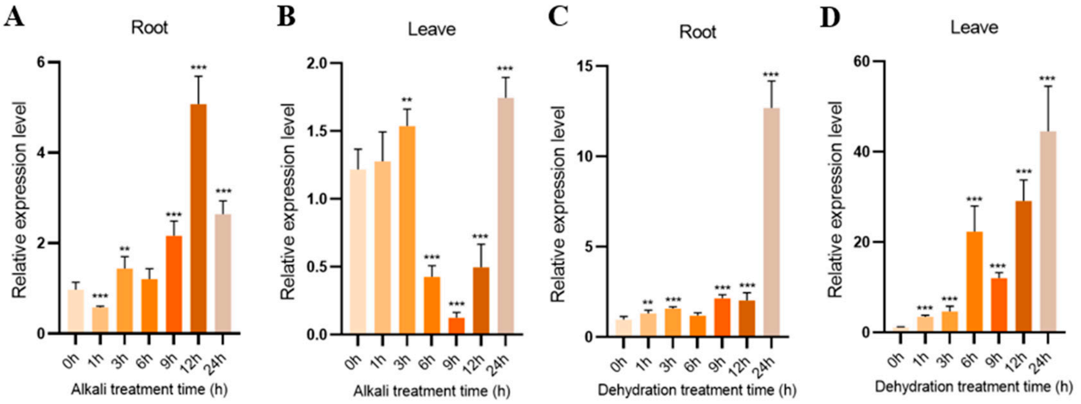

Figure S2. Analysis of expression levels of SaCHYR4 at different times after alkali-stress and drought-stress exposure. (A) Expression level of SaCHYR4 in roots at different times after alkali stress. (B) Expression level of SaCHYR4 in leaves at different times after alkali stress. (C) Expression roots of SaCHYR4 in leaves at different times after drought stress. (D) Expression level of *SaCHYR4* in leaves at different times after drought stress. **, $P < 0.01$, ***, $P < 0.001$.

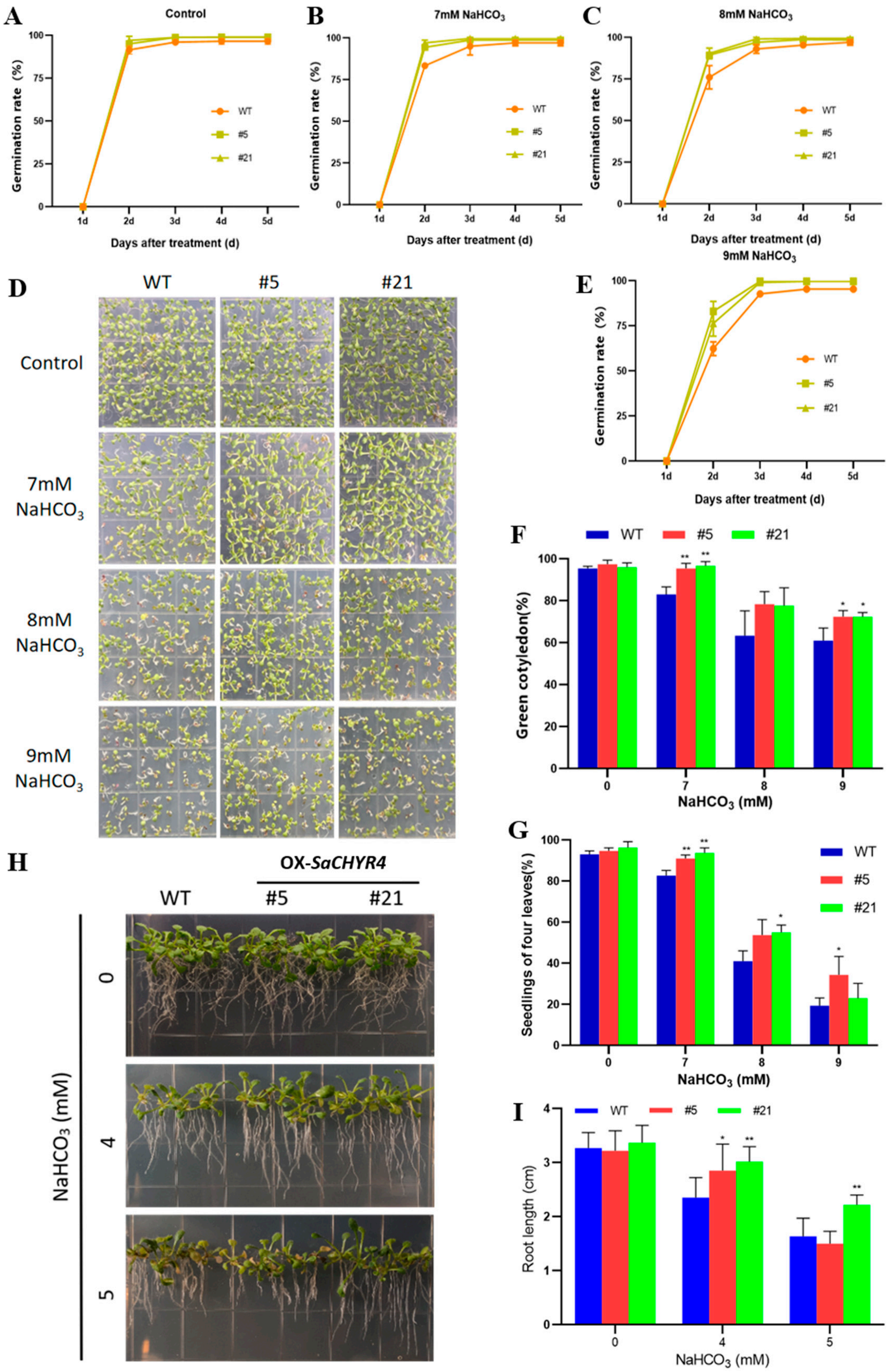

Figure S3. Effect of overexpression of *SaCHYR4* on the germination stage of *Arabidopsis thaliana* under alkali stress. Variables measured were germination rate: (A) control, (B) 7 mM

NaHCO3, (C) 8 mM NaHCO3, (E) 9 mM NaHCO3; (D) phenotypic state; (F) green cotyledon; (G) seedlings of four leaves; (H) phenotypic state; and (I) root length. *, P < 0.05; **, P < 0.01.

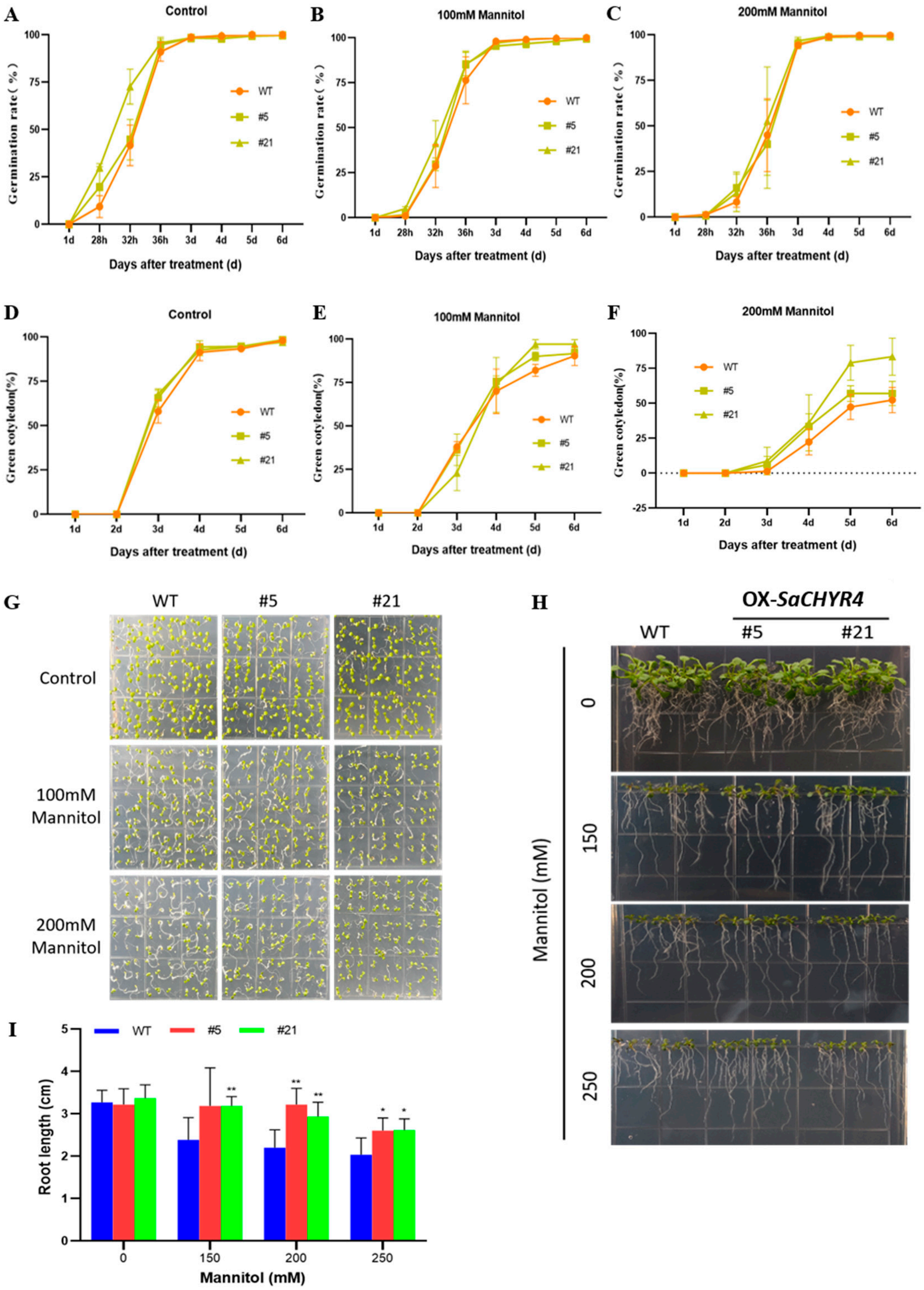

Figure S4. Effects of overexpression of *SaCHYR4* on the germination of *Arabidopsis thaliana* under simulated drought stress. Variables measured were germination rate: (A) control, (B) 100 mM mannitol, and (C) 200 mM mannitol; green cotyledon: (D) control, (E) 100 mM mannitol, and (F) 200 mM mannitol; Phenotypic status (G) germination stage (H) seedling stage; and (I) root length. *, P < 0.05; **, P < 0.01.
